# Bronchogenic Cyst of the Filum Terminale: A Case Report and Literature Review

**DOI:** 10.7759/cureus.83717

**Published:** 2025-05-08

**Authors:** Ali M Sumayli, Ahmed J Alzahrani, Mohamed M Aly, Mohamed A Mehrez

**Affiliations:** 1 Neurosurgery, King Khalid Hospital, Najran, SAU; 2 Neurosurgery, Security Forces Hospital, Riyadh, SAU; 3 Neurosurgery, Prince Mohamed Ben Abdelaziz Hospital, Riyadh, SAU

**Keywords:** bronchogenic cyst, case report, filum terminale cyst, intraspinal cyst, urinary retention (ur)

## Abstract

This case report delineates the case of a 35-year-old male patient who has experienced an acute onset of difficulty initiating and evacuating his bladder, with a nine-month background history of gradually progressive low back pain. The patient also had urinary urgency. The MRI spine revealed a well-defined intradural extra-medullary non-enhancing cyst at T12-L1 that measured 1.1 x 1.3 cm and resulted in significant cord compression. There was no other cutaneous, spinal cord, or vertebral abnormality associated. The patient underwent a T12-L1 laminectomy and a gross total excision of the spinal intradural extramedullary cyst, which was observed to be firmly attached to the filum terminale. The opaque fluid was obtained by puncturing the resected cyst. There was no change in the somatosensory evoked potential during the cyst resection. The patient observed a substantial improvement in his voiding habit on the first postoperative day and was discharged two days later. The histological examination verified the diagnosis of a bronchogenic cyst (BC) by demonstrating a benign cyst surrounded by ciliated pseudostratified columnar cells, dispersed Goblet cells, and underlying fibrous tissue. This case represents an example of intraspinal BCs, which are rare intradural extramedullary lesions, most commonly in the cervical or upper thoracic spine. They are slow-growing, and their clinical presentation is variable. Surgical resection remains the primary treatment for symptomatic cases, with gross total resection (GTR) preferred due to its superior outcomes in symptomatic relief and recurrence rates. However, complete resection is often challenging due to the cyst's ventral location or adherence to critical neural structures, emphasizing the importance of electrophysiological monitoring during surgery.

## Introduction

Endodermal cysts are developmental cysts that arise from the endoderm of the growing gastrointestinal tract (neuromeric cysts) or, in rare cases, the respiratory epithelium (bronchogenic cysts (BCs)) [1]. BCs are mostly found in the lung or mediastinum but rarely occur in cutaneous and subcutaneous tissues, the pericardium, the diaphragm, the abdomen, and the spinal cord [1]. Intraspinal BCs are exceedingly rare, with only 29 surgically treated cases reported in the literature. They are mostly located in the cervical spine in 51% of cases [2-9], followed by the lumbar spine in 17% in the intradural extramedullary compartment [2,3,9-11]. Intraspinal BCs are usually present with axial pain, radicular pain, or signs of spinal cord compression, where they present to the spine surgeon and require surgical intervention. To the best of our knowledge, there are no reports of filum terminale BC being surgically treated. Consequently, our objective is to document the initial instance.

## Case presentation

A 35-year-old male with a history of diabetes mellitus type 2 presented to the urology clinic with difficulty initiating voiding and emptying his bladder, fully associated with urinary urgency. The patient had a nine-month history of progressive low back pain. The patient denied fever, weight loss, leg pain, or walking difficulties. Urine analysis and culture showed no abnormality. Renal and bladder ultrasound ruled out stone or structural abnormality. Thereafter, the patient was referred to a neurosurgery clinic, where his neurological examination revealed no motor weakness in the lower limbs, a negative straight leg raising test, and no upper motor neuron signs. The patient was referred for magnetic resonance imaging (MRI) of the thoracic and lumbar spine with contrast based on a high index of suspicion for a spinal lesion due to progressive back pain and some urinary problems. There was no other cutaneous, spinal cord, or vertebral abnormality associated.

MRI findings

**Figure 1:**
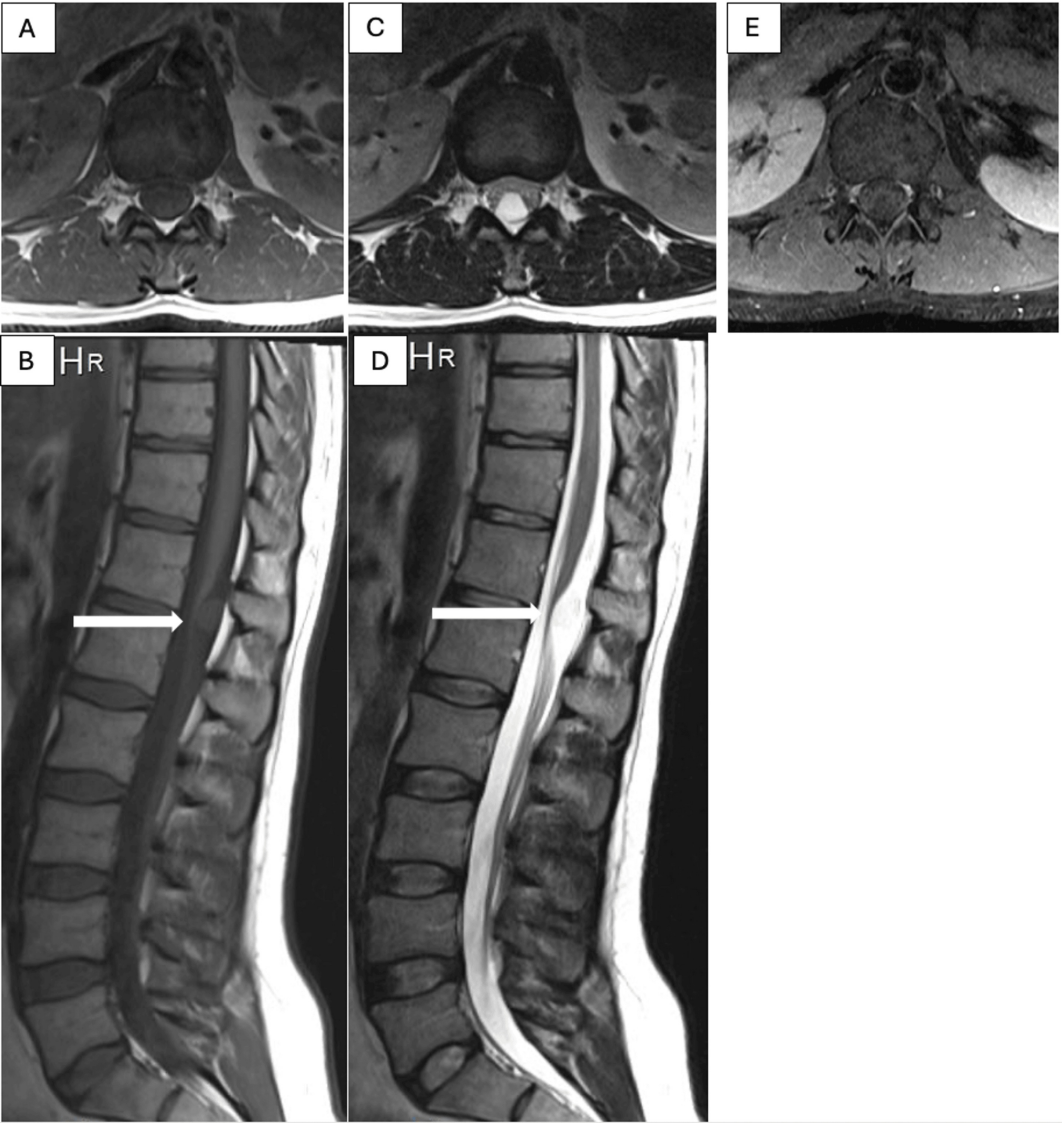
Magnetic resonance imaging (MRI) of the lumbar spine without and with contrast. MRI showing a 1.1 x 1.3 cm well-defined intradural extra-medullary lesion at the level of T12-L1, with isointense signal intensity on axial and sagittal T1WI (A, B), high signal intensity on axial and sagittal T2WI (C, D), and no significant enhancement post-contrast (E). T1WI: T1-weighted images, T2WI: T2-weighted images

The MRI spine showed a 1.1 x 1.3 cm well-defined intradural extra-medullary lesion at the level of T12-L1, causing significant cord compression. The cyst showed isointense signal intensity in T1-weighted images (T1WI) and high signal intensity in T2-weighted images (T2WI), with no significant enhancement post-contrast (Figures 1A-1D).

Surgical procedure

The patient underwent T12-L1 laminectomy and excision of the spinal intradural extramedullary cyst mass. A midline dural opening was done, and a round opaque cyst was seen tightly attached to the filum terminale. Direct stimulation of the attached filum terminale on both the rostral and caudal sides of the cyst revealed no action potentials. Then, the film attachment was coagulated and cut; then, the cyst was resected en bloc. Puncture of the resected cyst yielded opaque fluid. Microsurgical gross resection of the cyst could be performed. Somatosensory evoked potential revealed no change during the cyst resection.

Histopathology

**Figure 2:**
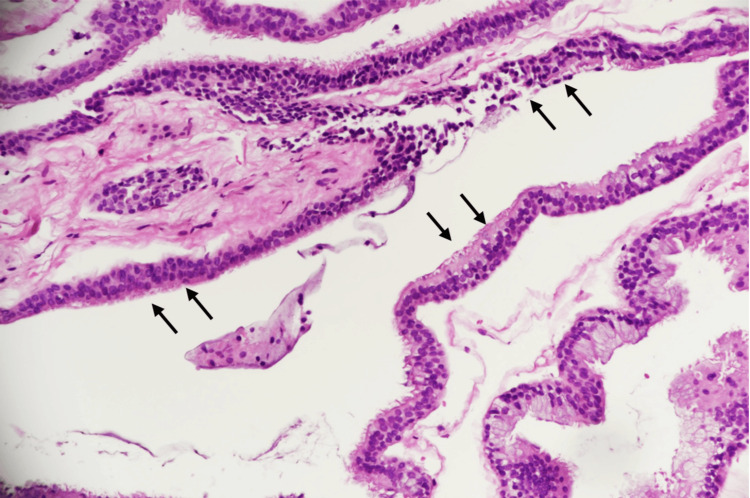
Low power magnification histopathology slide showing the cyst with underlying fibrous tissue (arrows).

**Figure 3:**
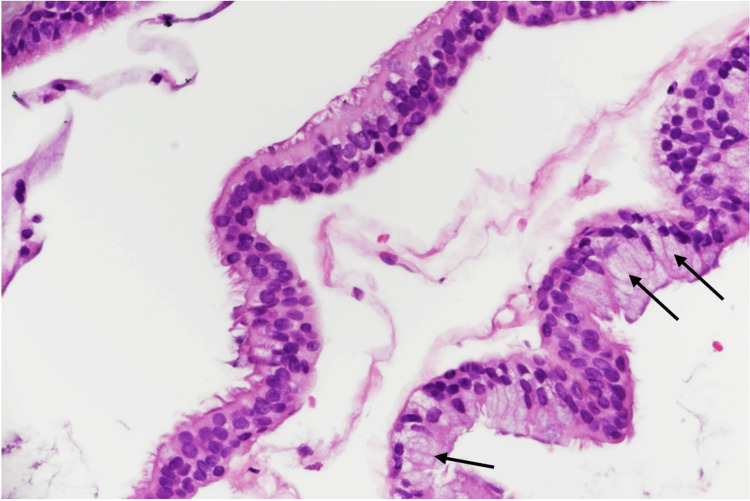
High magnification histopathology slide showing ciliated pseudostratified columnar cells with scattered goblet cells (arrows).

The histological examination confirmed the diagnosis of a bronchogenic cyst by revealing a benign cyst lined by ciliated pseudostratified columnar cells with scattered Goblet cells and underlying fibrous tissue. No evidence of cytological atypia or malignancy was seen (Figures 2, 3).

Postoperative course

The postoperative course was uneventful. Postoperative day 1, the patient noticed a significant improvement in his voiding habit, and he was discharged two days postoperatively. At the one-month follow-up appointment, the patient denied any back pain or voiding difficulty.

## Discussion

Intraspinal BC is typically intradural extramedullary lesions, most located at the cervical or upper thoracic spine level. Less common sites include the thoracolumbar junction, conus medullaris, craniocervical junction, and sacral spine. This case report details the successful surgical resection of a rare case of BC, the filum terminale. We are unaware of any prior reports of BC in the filum terminale.

BCs grow slowly owing to tight junctions between epithelial cells, limiting the expansion of the cyst. Intraspinal BCs are most frequently characterized by axial pain (63%) [2,3,5-10,12-16], followed by sensory disturbances (46%) [2,3,6-14,16,17], cord compression and paralysis (36%), [4,8,10,11,13,15-19], radicular pain (23%) [2,3,5,6,9,20], and sphincter dysfunction (13%) [11,12,15,16,18].

**Table 1:** Spinal bronchogenic cysts in previously published case reports and series. MRI, magnetic resonance imaging; T1WI, T1-weighted imaging; T2WI, T2-weighted imaging; T1-EN, T1 enhancement; F, female; NR, not reported; SBO, spina bifida occulta; GTR, gross total resection; LM, lumbosacral meningocele; PR, partial resection; M, male; Iso, isointense; Hyper, hyperintense; Hypo, hypointense; CCJ, craniocervival junction; CM, conus medullaries; MHI, mixed hyper and iso; SCT, spinal cord tethering; MHO, mixed hypo; MHE, mixed hyper; EPR, edge punctate reinforcement; STR, subtotal resection, KFA, Klippel-Feil anomaly.

Study	Age	Sex	Location (axial/longitudinal)	T1WI	T2WI	T1-EN	Anomaly	Surgery	Outcome
Yamashita et al., 1973	14 years	F	Dorsal/C6—C7	NR	NR	NR	SBO	GTR	Relieved
Ho and Tiel, 1989	21 years	F	Dorsal/C5-T3	NR	NR	NR	No	GTR	NR
Wilkinson et al., 1992	55 years	F	Dorsal/C3-C4	NR	NR	NR	No	PR	Relieved
Baba et al., 1995	16 years	M	Dorsal/C1	Iso	Hyper	NR	No	GTR	Relieved
Rao et al., 1999	18 years	M	Ventral/C2—C3	Hypo	Hyper	NR	No	GTR	Relieved
Baumann et al., 2005	41 years	NR	Dorsal to conus/T12—L1	NR	Hyper	No	SBO	PR	Refractory
Chongyi et al., 2008	28 years	M	Dorsal/L1	NR	Hyper	NR	No	PR	Relieved
Ko et al., 2008	5 months	F	Dorsal/S2	Hypo	Hyper	NR	SBO	GTR	Relieved
Arnold et al., 2009	20 years	M	Dorsal/T4	NR	Hyper	NR	No	GTR	Relieved
Yilmaz et al., 2009	17 years	M	Conus medullaris	NR	Hyper	NR	No	PR	Relieved
Solaroglu et al., 2014	50 years	F	Ventral/craniocervical junction	Iso	Hyper	No	No	GTR	Relieved
Chen et al., 2015	24 years	M	Dorsal/L4-L5	Iso	Hyper	No	LM	PR	Relieved
	34 years	M	Dorsal/craniocervical junction	Hypo	Hyper	No	No	PR	Relieved
	29 years	M	Ventral/T9-T10	Iso	Hyper	No	Scoliosis	GTR	Relieved
Zou et al., 2015	44 years	F	Dorsal/conus medullaries	MIHI	Hyper	NR	SCT	GTR	Relieved
Liu et al., 2015	55 years	M	Dorsal/T5-T7	MHO	MIHE	NR	No	PR	Relieved
Kandula et al., 2016	51 years	M	Dorsal/L3-L4	NR	Hyper	NR	No	GTR	Refractory
Ma et al., 2017	23 years	F	Ventral/C4-C7	Hypo	Hyper	No	No	PR	Relieved
	37 years	F	Ventral/C3-C6	Hypo	Hyper	No	No	PR	Relieved
	66 years	M	Dorsal to the conus/L1-L2	Hypo	Hyper	EPR	No	PR	Relieved
Lee et al., 2017	44 years	M	Dorsal/T12-L1	Hypo	Hyper	NR	No	GTR	Relieved
Dusad et al., 2017	18 years	M	Central/T3-4	Hyper	Hypo	NR	No	PR	Refractory
Weng et al., 2018	23 years	M	Dorsal/C3-C4	Hypo	Hyper	No	No	STR	Relieved
	15 years	M	Dorsal/L1-L2	MHI	MHI	No	No	STR	Refractory
	25 years	F	Ventral/C2—C4	Hypo	Hyper	No	No	GTR	Relieved
	41 years	F	Ventral/C4	Hypo	Hyper	No	No	STR	Relieved
	6 years	M	Ventral/C2-C5	Hypo	Hyper	No	Scoliosis	STR	Refractory
	36 years	F	Lateral/medulla oblongata—C2	Hypo	Hyper	No	No	GTR	Relieved
Wu., 2021	48 years	M	Septated ventrolateral C2-6	Hypo	Hyper	Nodular	KFA	PR	Neck pain diminished, numbness relieved but weakness, paresis

Microsurgical resection of symptomatic intraspinal BCs is necessary to acquire tissue for a conclusive histological diagnosis and to decompress neuronal components. Gross total resection (GTR) should be attempted as it is associated with a significantly higher rate of symptomatic relief and a lower rate of recurrence than partial resection (82% vs. 18% and 0% vs. 63%, respectively). However, GTR could be achieved in only 48% [1-5,7,11,12,14,15,17,19], while subtotal or partial resection in the remaining 52% [2,3,6,8-10,13,16,18,20]. The main culprit for subtotal resection was the ventral location or the adherence of the cyst to the spinal cord [3,18] or dorsal conus medullaris and the cauda equina [10,20]. In that context, the importance of electrophysiological monitoring in maximizing safe resection cannot be overemphasized.

BCs are believed to be developmental in origin. Nevertheless, the precise developmental mechanics of these cysts are still unknown. A possible mechanism is a faulty separation between the ectodermal and endodermal layers, which results in the inclusion of endodermal tissue within the ectodermal-derived spinal cord. Incomplete excalation of the chordal plate would lead to the persistence of the neuromeric canal and the associated anomalies of the notochord. Such as spina bifida occulta, scoliosis, spinal cord tethering, meningocele, and Klippel-Feil abnormality [1-3,7,8,15]. Due to the further differentiation of the endodermal layer into the digestive or respiratory tract, endodermal cysts may present with both types of histological features [1].

## Conclusions

Intraspinal BCs are rare, typically presenting as intradural extramedullary lesions, most commonly in the cervical or upper thoracic spine. They are slow-growing, and their clinical presentation is variable. Surgical resection remains the primary treatment for symptomatic cases, with GTR preferred due to its superior outcomes in symptomatic relief and recurrence rates. However, complete resection is often challenging due to the cyst's ventral location or adherence to critical neural structures, emphasizing the importance of electrophysiological monitoring during surgery.
